# Intra-site differential inhibition of multi-specific enzymes

**DOI:** 10.1080/14756366.2020.1743988

**Published:** 2020-03-25

**Authors:** Mario Cappiello, Francesco Balestri, Roberta Moschini, Umberto Mura, Antonella Del-Corso

**Affiliations:** a Department of Biology, Biochemistry Unit, University of Pisa, Pisa, Italy; b Interdepartmental Research Center Nutrafood “Nutraceuticals and Food for Health”, University of Pisa, Pisa, Italy

**Keywords:** Differential inhibitors, multi-specific enzymes, promiscuous enzymes, aldose reductase

## Abstract

The ability to catalyse a reaction acting on different substrates, known as “broad-specificity” or “multi-specificity”, and to catalyse different reactions at the same active site (“promiscuity”) are common features among the enzymes. These properties appear to go against the concept of extreme specificity of the catalytic action of enzymes and have been re-evaluated in terms of evolution and metabolic adaptation. This paper examines the potential usefulness of a differential inhibitory action in the study of the susceptibility to inhibition of multi-specific or promiscuous enzymes acting on different substrates. Aldose reductase is a multi-specific enzyme that catalyses the reduction of both aldoses and hydrophobic cytotoxic aldehydes and is used here as a concrete case to deal with the differential inhibition approach.

## Introduction

The concept of “one enzyme-one substrate”, along with the “lock and key” theory, is often referred to when presenting enzymes as extremely specific biocatalysts, but is easily disproven in reality. In fact, numerous multispecific enzymes, able to catalyse in the active site the same transformation of different substrate, are known^1–3^. Furthermore “promiscuous enzymes”^4^^,^^5^ are able to catalyse different reactions in the same active site, in addition to the reaction considered to be native. These reactions may occur through either the same or alternative mechanisms to those for the native reaction. The multipotency/broad-specificity of enzymes also appears in the so-called “moonlighting” behaviour of enzymes. In these cases, a different microenvironment in terms of the active site is recruited on the protein and a completely different function is conducted^6^^,^^7^. The relevance and the usefulness of these “unspecific behaviours” of enzymes (i.e. multi-specificity, promiscuity and moonlighting) have been widely considered in terms of both metabolic control and metabolism evolution and adaptation^8–12^. Enzyme multi-specificity and promiscuity are suggested to be key factors in evolution and adaptation, which is of great interest, in particular when considered in conjunction with the specific structural features of molecules to be recruited as substrates^13^^,^^14^, and may provide insights into the genesis of the native and physiologically relevant functions of the enzymes.

One aspect of the multi-specificity of enzymes, which to our knowledge has not been specifically addressed, concerns the feasibility, metabolic significance and usefulness of a differential inhibitory action directed at one or more, but not to all, of the reactions catalysed by a multi-specific enzyme. Our search for useful inhibitors of the multi-specific enzyme aldose reductase (AKR1B1) has led to our proposal of a new strategic approach for inhibiting the enzyme^15^. The aim of aldose reductase differential inhibition was to specifically target deleterious catalytic actions of the enzyme without interfering with its advantageous catalytic functions.

In this paper, we propose that this inhibition strategy can be generalised as a fine approach to controlling enzyme activity, and we provide an overview of the conditions determining or favouring differential inhibition.

## “Differential” inhibition

The term “differential inhibition” refers to the inhibition of a multi-specific or a promiscuous enzyme acting on one or more specific substrates, while the transformation of other substrates remains unaffected or affected at a reduced extent. Differential inhibition is defined as the difference between the percent of inhibition of the reaction that is more sensitive to the inhibitor and that of other substrate transformation in the same reaction conditions. A differential inhibition may also occur for multi-specific and promiscuous enzymes in the presence of classical (i.e. not differential) inhibitors. This can depend on the conditions in which the enzyme’s susceptibility to inhibition is tested, and is linked to the kinetic parameters characterising the transformation of the two substrates. An intuitive example is that of a Michaelian enzyme acting on two competing substrates *A* and *B*. For simplicity, we can consider that they are transformed with the same *k_cat_*, into the products *P* and *Q*, respectively. In the presence of a classical competitive inhibitor (*I*), differential inhibition is predicted to occur in a situation (condition *a*) in which the *K_M_* for the two substrates (i.e. *K_A_* and *K_B_*) are significantly different. In a typical scenario, the inhibition test is performed separately on each of the two substrates present in the assay mixture. Here, by using the simple Michaelis and Menten steady-state kinetic equation, imposing a 5-fold difference in *K_M_* values between the two substrates (i.e. *K_B_/K_A_* = 5), and considering [*I*] = *K_i_* and [*A*] = [*B*] = *K_B_,* a differential inhibition of *B* with respect to *A* of approximately 19% will result. This value increases to approximately 25% for a *K_B_/K_A_* ratio of 10. Similarly, a differential inhibition may occur in a situation (condition *b*) in which the *K_M_* of the two substrates are similar in magnitude, but their concentrations are significantly different. Here, if [*B*] is fixed at the *K_M_* value while [*A*] is kept at 5-fold the *K_M_* value and [*I*] = *K_i_*, a differential inhibition of approximately 19% between the *B* transformation and the *A* transformation can be predicted. This value increases to 25% if [*A*] is raised to 10-fold *K_M_*, or to approximately 26% if [*B*] is decreased to a value of *K_M_*/2.

When both substrates are simultaneously present in the above conditions, the equation rate must consider, in addition to the inhibitor, the reciprocal influence exerted by the two substrates^16^. Thus, for one of the cases described above (condition *a*) with *K_B_/K_A_* = 5, the relative equations describing products formation are:

(1) $$v_{p}=\frac{V_{A}[A]}{K_{A}\left(1+\frac{\left[B\right]}{K_{B}}\right)\left(1+\frac{\left[I\right]}{K_{i}}\right)+[A]}$$

(2) $$v_{q}=\frac{V_{B}[B]}{K_{B}\left(1+\frac{\left[A\right]}{K_{A}}\right)\left(1+\frac{\left[I\right]}{K_{i}}\right)+[B]}\;$$

Thus, essentially due to the different reciprocal influence of the two substrates, an increase in differential inhibition from 19%, which is assumed when each substrate is present alone, to 24% is predicted. Similarly, when the parameters describing the condition *b* (i.e. *K_A_=K_B_*, [*B*] = *K_M_* and [*A*] = 5 *K_M_*) are inserted into Equations 1 and 2, the differential inhibition of *B* versus *A*, predicted to be 19% when each substrate is present alone, increases to 24%. In the same conditions as above, but with [*B*] = *K_M_*/2, an increase in differential inhibition from 26% when each substrate is present alone to 29% when the two substrates are simultaneously present is predicted.

Although the above conditions are simply imposed to provide an immediate result, many other different combinations of kinetic parameters and concentrations of substrates and inhibitor can occur, which may enhance or attenuate the apparent inhibitory differential effect. When the different reaction conditions for the two substrates represent possible *in vivo* physio-pathological situations the resulting differential inhibition may be a useful modulatory action for controlling enzyme function.

## Intra-site differential inhibitors

In all the cases discussed above, we assume that the inhibitor we define as “classical” will intervene in the reaction irrespective of the substrate that the enzyme will transform. However, an inhibitory molecule may intervene differently in the transformation of two substrates when the interactions between the substrates and the active site are not the same. Thus, depending on the structural features of the substrates, different functional groups may be recruited and/or a different steric hindrance may result. This may well be the case for promiscuous enzymes, for which the two substrates undergo different reactions, thus enabling them to interact with different protein groups. In the most general case of multi-specific enzymes, which may or may not share the same pattern of functional groups that allow catalysis, the substrates may lead to a very specific range of interactions, particularly if they are significantly different in their structural features. This can then lead to a different interaction with the inhibitor. These conditions are the basis for a “mechanistic” generation of differential inhibition, in which the inhibitor is the active part of the phenomenon.

Thus, the simplest definition of a “complete” intra-site differential inhibitor (*DI*) is a molecule that can interfere specifically with the transformation of one or more substrates while leaving the transformation of one or more other substrates free to occur. Differential inhibitors, which allow the enzyme to act on one of the possible substrates, may modulate the functions associated with multi-specific activity^17–19^. Differential inhibition refers to multi-specific and promiscuous enzymes in which the catalytic action for all the substrates takes place at the same active site. On the other hand, the activity of moonlighting enzymes, which is associated with different active sites, can be modulated by the classical site-specific inhibition approach.

**Figure 1.**
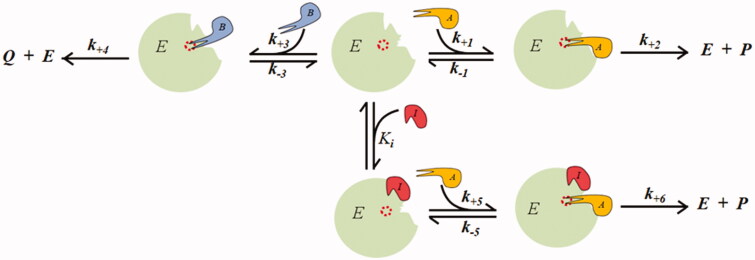
Competitive model of an intra-site differential inhibitor.

As classical inhibitors, differential inhibitors may intervene in catalysis through different models of action^16^. A schematic representation of a competitive inhibition model for a *DI*, the most adequate to express a differential inhibitory action, is given in Figure 1.

Here, only the transformation of the substrate *B* into the product *Q* is susceptible to inhibition, while the transformation of the substrate *A* into the product *P* is unaffected by the inhibitor. When analysed in steady state conditions, the inhibition model of Figure 1 can be described by two rate equations^16^. The generation rate of product *Q* is given by Equation 2, while Equation 3 gives the generation rate of product *P*; here, an apparent increase rather than a decrease in the reaction rate is observed in the presence of the *DI*.

(3) $$v_{p}=\frac{k_{+2}E_{T}\left[A\right]}{K_{A}\left(1+\frac{\frac{\left[B\right]}{K_{B}}}{1+\frac{\left[I\right]}{K_{i}}}\right)+\left[A\right]}$$

**Figure 2.**
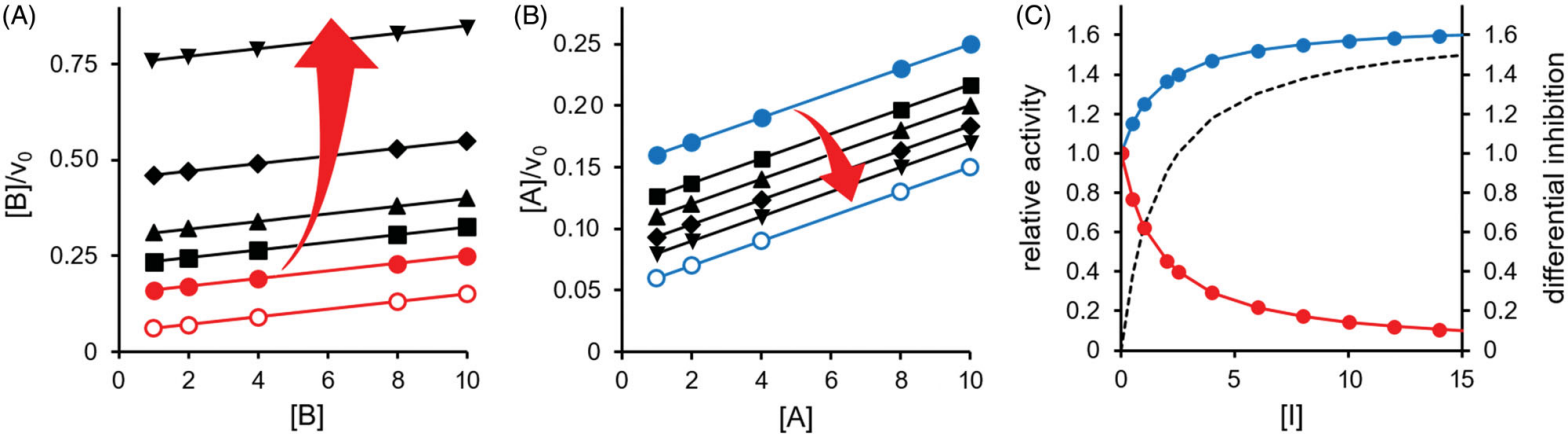
Predicted effect of a competitive *DI* on the transformation of two substrates catalysed by a multi-specific enzyme. The kinetic behaviour is described by the model reported in Figure 1 with the two substrates displaying the same *K_M_* (i.e. *K_A_* = *K_B_ = 5* concentration units) and the same *k_cat_* (100 time^−1^ units) and is represented by the Hanes–Wolff plot. Panel A: the transformation of substrate B, which is susceptible to inhibition, is analysed. The red open and closed circles refer to substrate *B* present alone and in the presence of *A* at a concentration equal to 2*K_M_*, respectively. Symbols ▪, ▲, ♦ and ▼refer to red closed circles in the presence of [I] equal to 1, 2, 3, and 5 times the *K_i_* value, respectively. The *K_i_* value is considered as *K_M_*/10. Panel B: the transformation of the substrate *A*, which is insensitive to direct inhibition, is analysed. Blue open and closed circles refer to substrate *A* alone and in the presence of *B* at a concentration equal to 2*K_M_*, respectively. Symbols ▪, ▲, ♦ and ▼ refer to blue closed circles in the presence of [I] equal to 0.5, 1, 2, and 5 times the *K_i_* value, respectively. The *K_i_* value is considered as *K_M_*/10. In Panels A and B, the increase in [I] is emphasised by the red arrows. *In* Panel C, the B rate transformation (red curve) and A rate transformation (blue curve) are reported as a function of the inhibitor concentration expressed as *K_i_* fold. The substrate concentrations were considered fixed at the 2K_M_ value and the *K_i_* value is considered as *K_M_*/10. The dotted line refers to the differential inhibition.

The differential action of the *DI* on the transformation of substrate *B* will relieve the competitive inhibitory action substrate *B* exerts on the transformation of substrate *A.* This gain in the *A* rate transformation will increase with the *DI* concentration. The direct inhibitory effect on the *B* transformation and the indirect enhancing effect on the *A* transformation exerted by a competitive *DI* acting in the presence of the two substrates are reported in Figure 2(A,B), respectively. The progressive increase of differential inhibition as a function of *DI* concentration observed when [*A*] = [*B*] = 2*K_M_* is reported in Figure 2(C).

The competitive inhibitor for substrate *B* may also be able to partially inhibit the transformation of the competing substrate *A*. The model in Figure 1 can also describe this, in which the ternary complex *EIA* evolves into a product with a kinetic constant *k_+6_* lower than *k_+2_*.

**Figure 3.**
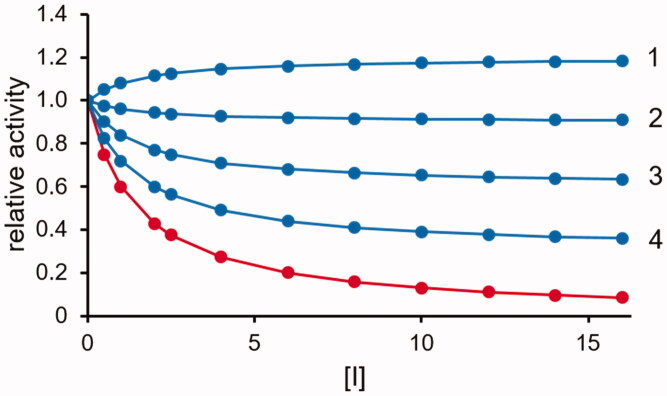
Effect of a competitive *DI* acting as a partial inhibitor of *A* transformation. The transformation rate of substrate *B* (red curve) and substrate *A* (blue curves) (see Figure 1) as a function of the inhibitor concentration (expressed as *K_i_* fold) is reported. The substrate concentration is considered fixed at the *K_M_* value (*K_A_* = *K_B_*). Curves 1, 2, 3 and 4 refer to *k_+6_* values equal to 0.8, 0.6, 0.4 and 0.2 times the *k*_+*2*_ value, respectively. The *K_i_* value is considered as *K_M_*/10.

The effect of this situation is that the differential inhibition is progressively reduced along with the increase in difference between *k_+6_* and *k_+2_*, as shown in Figure 3. As mentioned above, the competitive type of inhibition is the most effective and theoretically is the only model in which an inhibitor can be a “complete” differential inhibitor. Non-competitive inhibition requires the generation of a ternary complex *ESI.* This implies a reduction in the catalyst available for catalysis, which will also indirectly affect the reaction that is preserved. A differential inhibitory action, although incomplete, may also occur for non-competitive models of inhibition, depending on the kinetic parameters of the transformation of the two substrates.

**Figure 4.**
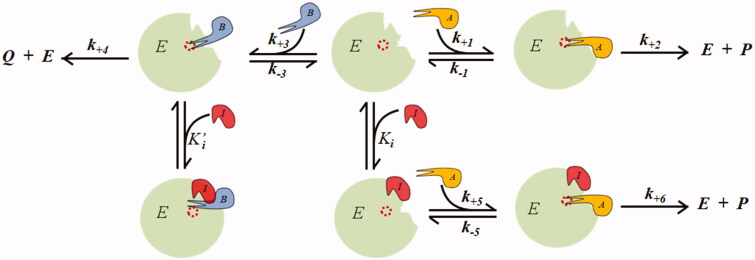
Non-competitive model of an intra-site differential inhibitor.

This can be rationalised through the model shown in Figure 4, in which the *DI* acts on the *B* transformation as a non-competitive inhibitor. Here, a steady state kinetic equation can be derived (Equation 4), which also describes the influence of the inhibitor on the reaction to be preserved (*A* transformation), which depends on the inhibitory features of the *B* transformation.

(4) $$v_{p}=\frac{(k_{+2}+\frac{\left[I\right]}{K_{i}}*k_{+6}){*E}_{T}}{\frac{K_{A}}{\left[A\right]}+\frac{K_{A}*\left[I\right]}{K_{i}*\left[A\right]}+\frac{\left[I\right]}{K_{i}}+\frac{K_{A}*\left[B\right]}{K_{B}*\left[A\right]}+\frac{K_{A}*\left[B\right]*\left[I\right]}{K_{B}*K_{i}^{'}*\left[A\right]}+1}$$

(5) $$v_{q}=\frac{k_{+4}*E_{T}}{\frac{K_{B}}{\left[B\right]}+\frac{K_{B}*\left[I\right]}{K_{i}*\left[B\right]}+\frac{\left[I\right]}{K_{i}^{'}}+\frac{K_{B}*\left[A\right]}{K_{A}*\left[B\right]}+\frac{K_{B}*\left[A\right]*\left[I\right]}{K_{A}*K_{i}*\left[B\right]}+1}$$

**Figure 5.**
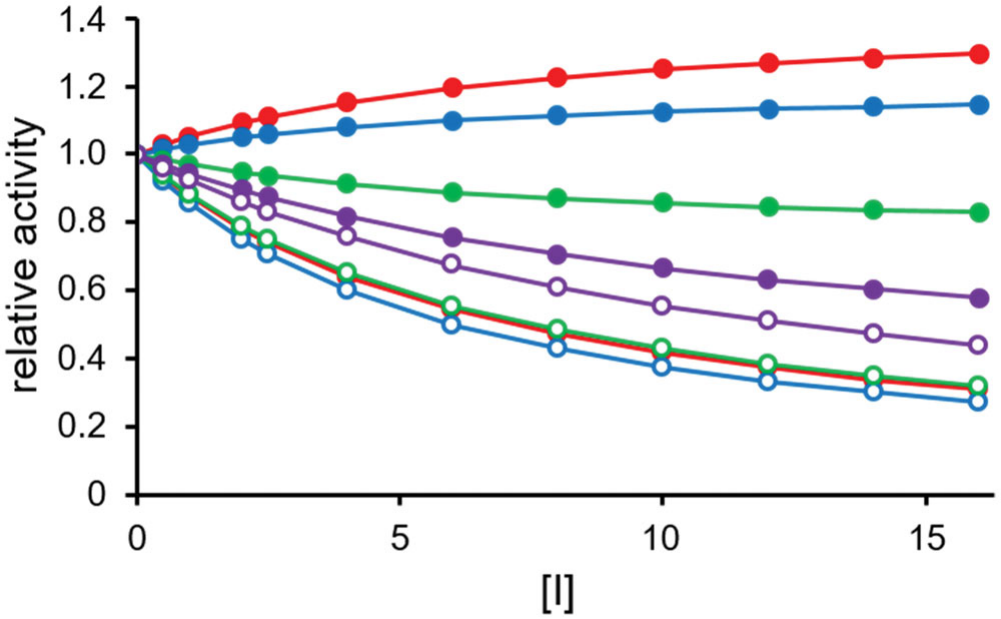
Effect of a non-competitive *DI*. The rate transformation of substrate *B* (open circles, see Equation (5) and substrate *A* (closed circles, see Equation (4) according to the model of Figure 4 are reported as a function of the inhibitor concentration (expressed as lowest *K_i_* fold). The substrates concentration is considered fixed at the *K_M_* value (*K_A_* = *K_B_*). The following combinations of inhibition constants are considered: red curves: *K_i_= K_M_*/10 and *K′_i_= K_M_;* blue curves *K_i_= K_M_*/10 and *K′_i_ = K_M_*/5*;* green curves *K_i_= K_M_*/5 and *K′_i_= K_M_*/10*;* violet curves *K_i_= K_M_* and *K′_i_= K_M_*/10.

Computer simulations of Equations 4 and 5 are reported in Figure 5. The *K′_i_/K_i_* ratio, which expresses the relative efficiency of the inhibitor in targeting the complex *EB* with respect to the free enzyme, clearly modulates the engagement of *A* in the inhibition process. Thus, the inhibitor progressively loses its character of *DI,* extending its action on the transformation of the substrate *A*.

The experimental measurements of the inhibition kinetic parameters that were independently conducted on the two substrates (as usually occurs in inhibitor screening) refer to “apparent” inhibitory constants (i.e. *^app^K_i_*, *^app^K′_i_*), whose relative values may lead to apparent incongruities. For example, if the experimental data indicate a mixed type of inhibition for substrate *B* and an uncompetitive inhibition for substrate *A*, as in neohesperidin dihydrochalcone (NHDC), which has been assessed as an inhibitor of the reduction of L-idose (substrate *B*) and HNE (substrate *A*) catalysed by AKR1B1^20^. The results of the analysis of substrate *B* shows that the inhibitor may target the free enzyme, and thus this should also result from the analysis of substrate *A.* However, the data suggest that the inhibitor targets only the *EA* complex. This apparently contradictory situation can be rationalised although not completely, in the following ways: (i) the measured apparent inhibitory constants can be regarded as true dissociation constants, in which the *K′_i_* for substrate *A* is significantly lower than the *K_i_* measured for substrate B (which is not the case for NHDC); and/or (ii) the measured constants are indeed “apparent” and the ternary complexes *EIA*, which is generated by adding *A* to the *EI* complex, and *EAI*, which is generated by adding *I* to the *EA* complex, are kinetically different and only *EIA* is able to evolve into products. Thus, once the potential *DI* has been identified through individual substrate analysis, it will be necessary to proceed analysing the simultaneous presence of the competing substrates for a conclusive evaluation of the differential inhibitory ability of the selected molecule.

*DIs* must exhibit more restrictive features than classical inhibitors. The strategic approach to searching for classical inhibitors is typically to disclose molecules able to specifically intervene on the target enzyme, and that interact with as many groups as possible at the active site. These are crucial for recognition and/or catalysis. This approach is often supported by molecular modelling and diffraction studies on the complex enzyme/inhibitor and is aimed at the improvement of both selectivity towards the target enzyme and inhibition potency. A very powerful inhibitor binding capacity at the active site is a very poor feature for a *DI* as it must limit the number of interactions, and for the substrate that is not expected to be affected it must preserve any possible interaction that can help enable its transformation. This consideration suggests that a significant inhibition potency cannot generally be an expected feature for a *DI*.

Different features in the bindings of the competing substrates may not enable the inhibitor to intervene only on the transformation of one of them. Therefore, it is possible that inhibition may indeed take place, although only partially or with different models of action, on the two different substrates. Thus, although molecules considered as inhibitors in the above examples cannot be defined as “complete” *DIs*, they still discriminate, at a mechanistic level, the different substrate molecules. This offers another instrument, which can be combined with the set of conditions in which the enzymatic reaction is operating, to elicit differential inhibition of one substrate with respect to the other.

## Aldose reductase differential inhibition

The multi-specific enzyme on which differential inhibition was first proposed as a potentially useful inhibition approach is AKR1B1 (EC 1.1.1.21). This enzyme is an aldo-keto reductase able to catalyse the NADPH-dependent reduction of numerous aldehydic compounds. AKR1B1 is usually presented as the first enzyme of the so-called polyol pathway, in which glucose is reduced by the enzyme to sorbitol, which is in turn transformed into fructose through a NAD^+^-dependent oxidation catalysed by sorbitol dehydrogenase^21^. Due to the relatively poor affinity the enzyme shows towards glucose^22–26^, it is debateable whether glucose can be considered as the native substrate for AKR1B1. However, although the polyol pathway is not a highly efficient metabolic route for glucose metabolism, it has been linked to the cellular osmotic control mediated by intracellular sorbitol levels^27^^,^^28^. The situation changes in hyperglycaemic conditions, where the flux of the pathway dramatically increases, thus making it a co-causative factor in the aetiology of secondary diabetic complications. The accumulation of sorbitol and the consequent osmotic unbalance, together with an alteration of the proper redox status of both NAD and NADP cofactors and the accumulation of the potent glycating agent fructose, lead to cell damage^29–32^. Thus, AKR1B1 has been extensively studied in terms of its inhibition^33–38^.

In addition to its involvement in the polyol pathway, AKR1B1 has been recognised as fulfilling a detoxifying role, as it can efficiently reduce various toxic hydrophobic aldehydes, such as alkanals and alkenals generated through membrane lipid peroxidation as a result of oxidative stress^39–42^. Glutathionylated alkenals, generated through the action of glutathione S-transferases^43^^,^^44^, are also substrates for AKR1B1^15,^^45^. These compounds, devoid of the reactive double bond and with more hydrophilicity than their un-glutathionylated counterparts, represent significant intermediates in the transformation of alkenals.^46^ Thus, the ability of AKR1B1 to catalyse their reduction appears to accomplish the detoxification. However, the reduction of 3-glutathionyl,4-hydroxynonanal (GSHNE), which is the glutathionyl adduct of HNE and a representative lipid peroxidative product, generates 3-glutathionyl-2,4-dihydroxynonane. This molecule has been reported to activate the NF-kB signalling cascade, thus promoting inflammation^47–49^. This may well represent the base of the anti-inflammatory action reported for a numbers of aldose reductase inhibitors (ARIs)^50^.

The multifaceted activities of AKR1B1 have antagonistic effects that depend on the substrate undergoing reduction, and drug development from ARIs has been relatively poor when compared to the *in vitro* discovery of selective and potent inhibitors of the enzyme. Thus, we propose a new strategy to approaching AKR1B1 inhibition^15^ and use terms such as “aldose reductase differential inhibitors” (ARDIs) and “intra site differential inhibition” to refer to molecules and conditions, respectively, which are able to determine an inhibition of the enzyme depending on the nature of the substrate the enzyme is working on. Within this frame, useful ARDIs include molecules able to intervene on glucose reduction, leaving unaltered, or less affected, the detoxifying activity of the enzymes towards hydrophobic aldehydes. Molecules able to differentially inhibit the reduction of GSHNE with respect to toxic hydrophobic aldehydes may be valuable, because of the inflammatory potential associated with GSHNE reduction.

Although a “complete” intra-site differential inhibitor as defined above has not as yet been envisaged for AKR1B1, molecules able to differentially inhibit aldoses reduction and/or GSHNE reduction versus HNE reduction have been proposed. The typical basic conditions to illustrate *in vitro* the differential inhibition of AKR1B1 mimic those occurring in a hyperglycaemic status, with the aldose substrates kept at the mM level, and the toxic aldehydes (i.e., HNE) or their glutathionyl-derivatives (i.e., GSHNE) kept at the µM level. D,L-glyceraldehyde, the most common substrate used in AKR1B1 inhibition studies, was also utilised as a substrate in differential inhibition studies. However, the evidence of incomplete inhibitory action exerted on the enzyme activity by aldose hemiacetals^51^^,^^52^, which cannot take place with a triose, suggested the use of an aldohexose as the substrate. Thus, L-idose, an epimer of D-glucose at C5, with a free aldehyde form approximately 80 times higher than what was observed for glucose, was chosen as elective substrate for inhibition studies^53^. Finally, the problem of poor solubility of molecules often encountered in inhibition studies of AKR1B1 was overcome by using a proper aqueous cocktail of either methanol or dimethyl sulfoxide, after evaluating the limits of their suitability for the enzyme assay^20^. In these conditions, supported by kinetic analysis of the inhibitory action towards the different substrates, a number of molecules, coming both from organic synthesis and from natural sources, were identified as having differential inhibitory abilities.

**Figure 6.**
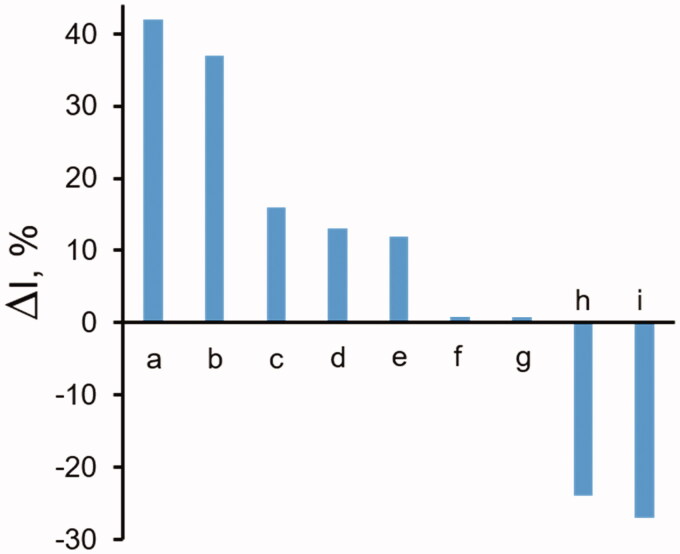
Differential inhibition of AKR1B1 by synthetic and natural compounds. The bars refer to the differential inhibition between aldoses reduction and HNE reduction. The letters along the bars refer to: a: D-glyceramide^15^; b: D-gluconamide^15^; c: pyrazol[1,5-a]pyrimidine derivative (compound 5e^52^); d: soyasaponin^55^; e: NHDC^20^; f: sorbinil^52^; g: epalrestat^52^; h: [1,2,4]oxadiazol-5-yl-acetic acid derivative (compound 9^15^); i: Saccharin derivative (compound 16^15^).

Thus, chemically synthesised compounds such as D-glyceramide or D-gluconamide were found to be inhibitors of the bovine lens enzyme^15^, and differentially inhibit the glyceraldehyde reduction versus the HNE reduction. D-glyceramide was also differentially active towards GSHNE and, to a lesser extent, towards D-glucose reduction. A differential inhibitory action on the human recombinant AKR1B1 towards L-idose and GSHNE reduction versus HNE reduction was also reported for some pyrazolepyrimidine derivatives^54^. The differential effects of selected compounds on AKR1B1 activity, derived from either organic synthesis or from natural sources, are summarised in Figure 6. Data relative to classical ARIs are also included as negative controls. Examples of molecules preferentially inhibiting HNE reduction over glyceraldehyde reduction are also reported.

Natural sources represent an almost limitless resource of biomolecules that can potentially affect the activity of enzymes, and this obviously also applies to the present inhibitory approach. Components exhibiting differential inhibitory ability were found in the fractionation of methanolic extracts of some edible vegetables^55^. The Zolfino bean, a variety of *Phaseolus vulgaris*, has been revealed to be a very promising source of ARDIs. After the bean was characterised in terms of classical AKR1B1 inhibitory activity^56^, components identified as soya saponins were isolated and shown to differentially inhibit to some extent L-idose reduction versus HNE reduction^57^.

## Conclusions

The ability of enzymes to convert molecules that are structurally different through the same type of reaction, and the possibility of the same enzyme catalysing different reactions, may require the modulation of the enzyme activity depending on the substrate undergoing transformation. Thus, the “differential inhibitors” analysed in this work are the instruments that can specifically address the catalytic potential of multi-specific or promiscuous enzymes towards one of their possible multiple functions.

Although no “complete” differential inhibitors of the multi-specific enzyme AKR1B1 have as yet been disclosed, knowledge of the interactive features and structural restraints a molecule needs to act as an ARDI is increasing. Studies conducted up to now on ARIs, in which ARDIs may have been disregarded, can be revisited, as an advantageous starting point to further this novel inhibition approach. Positive results for this enzyme target will also more generally confirm the potential of this intriguing inhibition approach for multi-specific or promiscuous enzymes.
